# Presence of tuberculosis symptoms among HIV-positive men who have sex with men (MSM) in Zimbabwe

**DOI:** 10.1186/s12981-024-00605-8

**Published:** 2024-03-28

**Authors:** Munyaradzi Mapingure, Innocent Chingombe, Tafadzwa Dzinamarira, Brian Moyo, Chesterfield Samba, Delight Murigo, Owen Mugurungi, Elliot Mbunge, Rutendo Birri Makota, Grant Murewanhema, Godfrey Musuka

**Affiliations:** 1 ICAP in Zimbabwe, Harare, Zimbabwe; 2 AIDS and TB Programmes, Ministry of Health and Child Care, Harare, Zimbabwe; 3 GALZ, Harare, Zimbabwe; 4 Department of Computer Science, Faculty of Science and Engineering, University of Eswatini, Kwaluseni, Eswatini; 5 Department of Biological Sciences and Ecology, University of Zimbabwe, Harare, Zimbabwe; 6 Unit of Obstetrics and Gynaecology, Faculty of Medicine and Health Sciences, University of Zimbabwe, Harare, Zimbabwe; 7 International Initiative for Impact Evaluation, Harare, Zimbabwe

**Keywords:** HIV, Tuberculosis symptoms, Case finding, MSM

## Abstract

We conducted secondary data analysis using a biobehavioral survey dataset of 1538 MSM from Zimbabwe. Survey participants were screened for the four symptoms suggestive of tuberculosis infection using the WHO TB screening algorithm. Results: All participants experienced at least one symptom suggestive of tuberculosis. 40% of HIV-positive MSM reported having had a cough in the last month and 13% of them experienced unexpected weight loss. The prevalence of experiencing any of the four TB symptoms amongst HIV-positive MSM was 23%.

**Contribution**

There is an urgent need for active TB case finding and treatment amongst HIV-positive MSM in Zimbabwe. Clinicians will need to ensure that MSM who need TB testing receive it timeously.

## Background

Despite being both preventable and treatable, TB remains an important public health problem in all countries and age groups [1]. Globally and in sub-Saharan Africa, tuberculosis is a major cause of mortality and morbidity [2, 3]. Being HIV-positive without viral load suppression is a predisposing factor for TB infection, amongst MSM. TB prevalence surveys in resource-poor countries consistently show a high burden of patients with TB disease who remain undiagnosed and are thus infectious to others [4]. Improving international case detection for TB also contributes to achieving health-related global goals [5]. HIV prevalence is declining in Zimbabwe but remains one of the highest in sub-Saharan Africa at 11.6% among the general population [6] and is higher at 23.4% among men who have sex with men (MSM) [7].

Eliciting of symptoms is an essential step in the diagnosis and care of presumptive MSM tuberculosis patients [8]. MSM are among the critical populations identified to be at higher risk of tuberculosis infection and disease [9]. However, there is limited TB data among MSM [10, 11].

## Methods

Data from our study on HIV and STI biobehavioral surveys (BBS) among MSM in Zimbabwe [7] was used to determine the prevalence of tuberculosis (TB) symptoms amongst HIV-positive MSM. Data were collected from 1538 individual MSM in the Zimbabwe cities of Harare and Bulawayo.

The study was a cross-sectional BBS using respondent-driven sampling (RDS). RDS is a peer-referral sampling methodology designed for data collection among hard-to-reach populations. Consenting participants completed a questionnaire on sociodemographics, sexual identity and history, sexual behaviour, mental health, alcohol and drug use, HIV testing services, stigma and violence, social cohesion, health services and TB. As part of the study, participants were screened for the four symptoms suggestive of tuberculosis infection using the WHO TB screening algorithm, and to our knowledge, this is the first and only BBS in Zimbabwe to have done so. After being tested for HIV, participants were also screened for tuberculosis (TB) symptoms. Participants who screened positive for TB symptoms were offered the option of being driven that day to a designated MSM-friendly referral healthcare facility that offers MSM-friendly HIV and other related services. Additionally, a peer escort to accompany them was provided. If the participant declined, they were given a referral card for the clinic that offers MSM-friendly HIV and related services. STATA statistical package version 17 was used for statistical analysis. The study received ethical clearance from the national institutional review board, the Medical Research Council of Zimbabwe # (MRCZ/A/2156).

## Results

In terms of demographics, the HIV-positive MSM were older than the negative, with Mean (Standard deviations) of 31.8 (9.2) and 26.4 (7.9) years respectively, *p* = 0.001. The majority (> 96%) were Black Africans, who had primarily attained (> 70%) secondary education. Close to 40% were unemployed, see Table 1. All participants experienced at least one symptom suggestive of tuberculosis, with prevalences of 7%, 4%, 7%, and 1% for persistent cough, night sweats, unexpected weight loss and fever respectively. Having had a cough in the last month was higher among HIV-positive MSMs compared to those not affected with HIV, 40% vs. 5%, *p* = 0.001. The same was the case with experiencing unexpected weight loss, 13% vs. 6%, *p* = 0.001 and experiencing a fever in the last month, 3% vs. 1%, *p* = 0.037. The prevalence of experiencing any of the four TB symptoms was 13%, and this prevalence was higher among the HIV-positive MSM compared to those who are not infected with HIV, 23% vs. 11%, *p* = 0.001. Three per cent reported that they had been exposed to a person with TB in the last 12 months.

**Table 1:** Baseline demographics and comparison of prevalence of TB symptoms among HIV Positive and HIV-negative MSM

Variable	HIV Positive	HIV negative	*P*-Value
***Demographics***			
Age in years			
Mean (Standard Deviation)	31.8 (9.2)	26.4 (7.9)	0.001
Ethnic Group			
Black African Other races	327 (96.2) 13 (3.8)	1,151 (98.3) 20 (1.7)	0.035
Education Level			
None None and Primary Secondary Tertiary Vocational	57 (4.9) 827 (70.6) 226 (19.3) 61 (5.2)	25 (7.4) 245 (72.1) 55 (16.2) 15 (4.4)	0.222
Employment Status			
Self-employed Employed full time Employed part-time Full-time student Retired Unemployed and unemployed	103 (30.3) 59 (17.4) 35 (10.3) 20 (5.9) 123 (36.2)	262 (22.4) 149 (12.7) 123 (10.5) 184 (15.7) 453 (38.7)	0.001
***TB Symptoms***			
In the last month, have you had a cough			
Prevalence	44/ 340 (40%)	62/1171 (5%)	0.001
In the last month, have you had night sweats?			
Prevalence	17/340 (5%)	37/1171 (3%)	0.108
In the last month, have you had any unplanned weight loss?			
Prevalence	43/340 (13%)	67/1171 (6%)	0.001
In the last month, have you had a fever or a “hot body”?			
Prevalence	9/340 (3%)	13/1171 (1%)	0.037
Experienced at least one of the four TB symptoms			
Prevalence	77/340 (23%)	125/1171 (11%)	0.001
In the past 12 months, have you been exposed to someone with TB?			
Prevalence	14/340 (4%)	39/1171 (3%)	0.487

## Discussions

A systematic review by Divala and others [8] shows that invariably, tuberculosis symptom screening is a critical entry point for the diagnosis of the disease [12–15]. Additionally, there is a need for more accurate screening tools, that remain accessible and low-cost for countries with high tuberculosis burden.

Some individuals wait for the spontaneous resolution of TB symptoms, and most do not seek healthcare help [15]. The biological and epidemiological interconnectedness of TB and HIV has generated a new form of stigma, TB–HIV stigma, which sometimes makes people afraid to go for TB symptoms as this would be perceived to be HIV-related TB.

We note that there is limited information on TB among critical populations such as MSM, and the findings in this letter are crucial for understanding TB symptoms among MSM. There is a need for ongoing campaigns to encourage people to seek healthcare help when experiencing TB symptoms so that we do not have infectious patients who remain undiagnosed and are thus contagious to others. Targeted TB screening is warranted, especially among key populations, resulting in a greater yield of identifying TB cases, which aligns with the International Standards for Tuberculosis Care.

Tuberculosis symptom screening has limitations. However, there is a need to increase the frequency of when it is offered to MSM to enhance the opportunity of identifying those individuals in need of further investigations and treatment. Additionally, in Zimbabwe MSM experience considerable amounts of stigma and discrimination at most health facilities due to cultural imperatives as noted in the formative survey which was part of the BBS survey [7]. For Zimbabwe to ensure its health for all targets are met timeously there is a need to ensure that no group is left behind, including MSM and other key populations. A key intervention by the Zimbabwe Ministry of Health will be to train its healthcare workers in the provision of key population-friendly services and non-discriminatory behaviour.

Non-governmental organisations in Zimbabwe provide principally HIV-related services to MSM in secluded locals to avoid harassment from the police and security forces [7]. It will be also key to provide tuberculosis-related screening to those MSM using such services. Those individuals who screen positive for tuberculosis should immediately be provided with tuberculosis diagnostic tests and those testing positive should be immediately linked to care.

## Abbreviations

BBS: Biobehavioral Survey
HIV: Human Immunodeficiency Virus
MSM: Men who have Sex with Men
RDS: Respondent Driven Sampling
TB: Tuberculosis
