# Correction: Presence of tuberculosis symptoms among HIV-positive men who have sex with men (MSM) in Zimbabwe

**DOI:** 10.1186/s12981-024-00617-4

**Published:** 2024-04-29

**Authors:** Munyaradzi Mapingure, Innocent Chingombe, Tafadzwa Dzinamarira, Brian Moyo, Chesterfield Samba, Delight Murigo, Owen Mugurungi, Elliot Mbunge, Rutendo Birri Makota, Grant Murewanhema, Godfrey Musuka

**Affiliations:** 1 ICAP in Zimbabwe, Harare, Zimbabwe; 2 AIDS and TB Programmes, Ministry of Health and Child Care, Harare, Zimbabwe; 3 GALZ, Harare, Zimbabwe; 4 Department of Computer Science, Faculty of Science and Engineering, University of Eswatini, Kwaluseni, Eswatini; 5 Department of Biological Sciences and Ecology, University of Zimbabwe, Harare, Zimbabwe; 6 Unit of Obstetrics and Gynaecology, Faculty of Medicine and Health Sciences, University of Zimbabwe, Harare, Zimbabwe; 7 International Initiative for Impact Evaluation, Harare, Zimbabwe

**Correction to: AIDS Research and Therapy (2024) 21:18**

10.1186/s12981-024-00605-8.

In this article [1], the statement in the Funding information section was incorrectly given as ‘The study was not funded’ and should have read ’ The secondary data analysis was not funded’.

## References

[1] Mapingure M, Chingombe I, Dzinamarira T, Moyo B, Samba C, Murigo D, Mugurungi O, Mbunge E, Makota RB, Murewanhema G, Musuka G. Presence of tuberculosis symptoms among HIV-positive men who have sex with men (MSM) in Zimbabwe. AIDS Res Ther. 2024;21(1):18. 10.1186/s12981-024-00605-8 PMC10979552 38549087

